# Direct quantitative detection for cell-free miR-155 in urine: a potential role in diagnosis and prognosis for non-muscle invasive bladder cancer

**DOI:** 10.18632/oncotarget.6487

**Published:** 2015-12-02

**Authors:** Xin Zhang, Yanli Zhang, Xinfeng Liu, Aiju Fang, Jinfeng Wang, Yongmei Yang, Lili Wang, Lutao Du, Chuanxin Wang

**Affiliations:** ^1^ Department of Clinical Laboratory, Qilu Hospital, Shandong University, Jinan 250012, China; ^2^ Department of Clinical Laboratory, Traffic Hospital of Shandong Province, Jinan 250031, China; ^3^ Department of Pathology, Traffic Hospital of Shandong Province, Jinan 250031, China

**Keywords:** cell-free, miR-155, urine, non-muscle invasive bladder cancer, biomarker

## Abstract

High recurrence rates of non-muscle invasive bladder cancer (NMIBC) in patients require lifelong testing and monitoring. The aim of this study is to develop a simplified RT-qPCR method (RT-qPCR-D) which directly quantifies cell-free miR-155 in urine without RNA extraction, and assess it as a potential tool in NMIBC detection. A pilot study including 60 urine samples was used to investigate the feasibility of RT-qPCR-D in detecting cell-free miR-155. Then, miR-155 levels were quantified in a large independent cohort of urine from 162 NIMBC patients, 76 cystitis patients, and 86 healthy donors using the RT-qPCR-D method. Changes of cell-free miR-155 before and after operation were also analyzed in 32 NIMBC patients. In pilot study, we found a significant linear association between RT-qPCR and RT-qPCR-D in urinary miR-155 detection. Both methods showed cell-free miR-155 were significantly increased in NMIBC patients, and could reflect their expression in tissues. Then, the increased expression of cell-free miR-155 was successfully validated in 162 NIMBC patients when compared with cystitis patients and healthy donors. Moreover, it distinguished NMIBC patients from others with 80.2% sensitivity and 84.6% specificity, which was superior to urine cytology. Cell-free miR-155 correlated with NMIBC stage and grade, and was an independent factor for predicting recurrence and progression to muscle invasion. In addition, cell-free miR-155 was significantly decreased after NMIBC patients underwent transurethral bladder resection. In conclusion, detection of cell-free miR-155 in urine using RT-qPCR-D is a simple and noninvasive approach which may be used for NMIBC diagnosis and prognosis prediction.

## INTRODUCTION

Worldwide, bladder cancer is the second most common malignancy of the urinary system and approximately 75% of newly diagnosed cases present non-muscle invasive bladder cancer (NMIBC) that is confined to the epithelium or subepithelial connective tissue [1, 2]. Despite receiving transurethral resection of the bladder and relevant control regimens, NMIBC still recur frequently and 10–15% cases will progress to muscle invasion with greater mortality [3], often requiring patients to undergo lifelong detection and monitoring. Cystoscopy is the gold standard for NMIBC surveillance and has high diagnostic accuracy, but is invasive, expensive and has low patient acceptance. While cytology has the advantage of being non-invasive, it provides diagnostic accuracies in the range of 11-76% and lacks sensitivity to detect low-grade disease [4]. Therefore, there is an urgent clinical need to develop new strategies for improved NMIBC detection.

Increasing publications have documented microRNAs (miRNAs), a class of non-coding RNAs composed of approximately 22 nucleotides, can act as tumor suppressors or oncogenes, the dysregulation of which are associated with cancer development and progression [5]. Recently, miRNAs have shown high stability in various types of body fluids, which allow for noninvasive analyses and, subsequently, demonstrate their potential as tumor markers [6, 7]. For example, use of a miRs-135b/15b/1224-3p panel isolated from urine could find 94.1% of symptoms suggestive of urothelial carcinoma, while decreasing invasive cystoscopy rates by 26% [8]. Urine-based miRNAs expression signatures were strong diagnostic and prognostic indicators for bladder cancer [9]. miR-155 is known as a typical multifunctional miRNA and is one of the most potent oncogenic miRNAs [10]. Recently, miR-155 has been found to be overexpressed in a wide array of malignant tumors (e.g. lymphomas [11], papillary thyroid carcinoma [12], hepatocellular carcinoma [13], non-small cell lung cancer [14], breast cancer [15]), often reflecting the presence of cancer, as well as staging, prognosis or treatment outcomes. Nonetheless, there is little known about cell-free miR-155 and further research is needed to investigate its role in NMIBC.

In this study, we systematically investigated the expression of cell-free miR-155 from urine supernatants in a two-phase study. In the pilot phase, we developed a reverse transcription quantitative real-time PCR (RT-qPCR) approach that was directly applied to urine supernatants (RT-qPCR-D) to quantify miRNAs without RNA extraction, which simplified the procedure. Then, we determined whether urinary miR-155 levels reflected the disease status as presented by NMIBC tissues using matched urine and tissue samples. In the validation phase, the clinical significance of cell-free miR-155 as a potential biomarker for the diagnosis and prognosis of NMIBC patients was evaluated using the established RT-qPCR-D method in a large, independent cohort. In addition, changes of cell-free miR-155 before and after operation were also analyzed in NIMBC patients underwent transurethral bladder resection.

## RESULTS

### PCR standard curve of U6, RNU48 and miR-155

**Figure 1:**
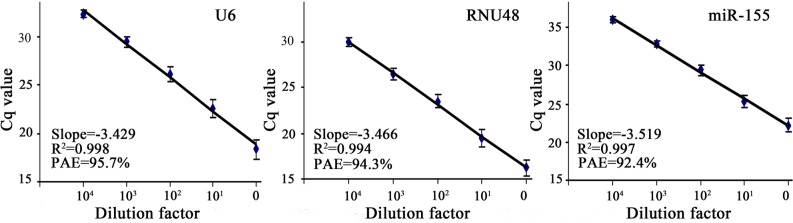
Standard curves for U6, RNU48 and miR-155 Cq value is the threshold cycle of PCR at which fluorescence is detectable. The horizontal axis (0, 10^1^, 10^2^, 10^3^, and 10^4^) is a series of 10-fold dilutions of cDNA from T24 bladder cancer cell line. PAE represents primer amplification efficiency.

As shown in Figure 1, the standard curves of reference genes (U6, RNU48) and miR-155 were generated from 10-fold serial dilutions of template (cDNA coming from T24 bladder cancer cell line, which expressed U6, RNU48 and miR-155) and the corresponding quantification cycle (Cq) values of U6, RNU48 and miR-155. The correlation coefficients (R^2^) between Cq value and serial dilutions of cDNA were all above 0.99. Primer amplification efficiency (PAE) of U6, RNU48 and miR-155 calculated from the slope of respective standard curve was 95.7%, 94.3% and 92.4%.

### Comparison between RT-qPCR-D and RT-qPCR in detecting urine cell-free miR-155

**Figure 2:**
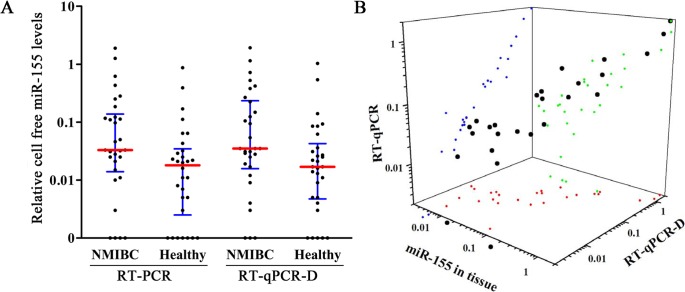
miR-155 expression in pilot phase **A.** Comparison of cell-free miR-155 levels among 30 NMIBC patients and 30 healthy detected by RT-qPCR-D and RT-qPCR. **B.** Correlation analyses between miR-155 expression in tissues and cell-free miR-155 levels in matched urine detected by RT-qPCR or RT-qPCR-D. Red, green, and blue dots represent the projection of miR-155 levels in NMIBC tissue, and the matched urine detected by RT-qPCR and RT-qPCR-D methods, respectively.

A pilot study including 60 urine samples was used to compare the two techniques (RT-qPCR-D and RT-qPCR) in detecting cell-free miR-155. The median relative concentrations (interquartile range) of miR-155 detected by both methods were shown in Table 1. Both assays measured levels of cell-free miR-155 in urine supernatants that were significantly increased in NIMBC patients in comparison to healthy donors (both at *P* < 0.001; Figure 2A). Moreover, there was a significant linear association between both assays (r = 0.956, *P* < 0.001; Figure 2B).

**Table 1:** Characteristics and cell-free miR-155 levels of subjects

	Healthy	Cystitis	NIMBC
**Pilot phase**			
case	30	/	30
Gender (Male/Female)	23/7	/	23/7
Age(y)^a^	65(41-81)	/	64(42-82)
miR-155 with RT-qPCR^a^	0.017 (0.005-0.043)	/	0.035 (0.016-0.136)
miR-155 with RT-qPCR-D^a^	0.018 (0.003-0.035)	/	0.033 (0.014-0.138)
**Validation phase**			
case	76	86	162
Gender (Male/Female)	61/15	67/19	126/36
Age(y)^a^	66(36-87)	64(35-82)	67(38-85)
miR-155^a^	0.010(0.002-0.017)	0.014(0.005-0.023)	0.044(0.027-0.144)

Abbreviations: NMIBC, non-muscle invasive bladder cancer; RT-qPCR, reverse transcription quantitative real-time PCR; RT-qPCR-D, RT-qPCR approach directly applied in urine supernatants.

a Data are presented as median (interquartile range).

The coefficient of variations (CV), including intra-assay CV and inter-assay CV, were used to compare the stability of two methods. Intra-assay variation was done by using 3 urine samples (covering the lower, median and upper miR-155 concentration) with triplicate measurement in a single PCR run. And the same samples were detected in 3 independent PCR runs for inter-assay CV calculation. As shown in Table 2, the CVs determined from the RT-qPCR-D intra-assay and inter-assay were slightly lower than RT-qPCR values, but this difference was not statistically significant (*P* > 0.05).

**Table 2:** Variability between RT-qPCR and RT-qPCR-D in detecting urinary cell-free miR-155

**Sample**	**intra-assay CV(%)**		**inter-assay CV(%)**	
	**RT-qPCR**	**RT-qPCR-D**	**RT-qPCR**	**RT-qPCR-D**
Sample 1	7.5	6.5	9.0	7.0
Sample 2	8.6	6.9	10.4	7.9
Sample 3	10.4	9.1	12.4	9.4
Mean value	8.8	7.5	10.6	8.1
Standard deviation	1.5	1.4	1.7	1.2

Abbreviations: CV, coefficient of variation; RT-qPCR, reverse transcription quantitative real-time PCR; RT-qPCR-D, RT-qPCR approach directly applied in urine supernatants.

### Association of miR-155 expression in urine and tissues of NIMBC patients

To determine whether urine levels of cell-free miR-155 reflect changes in NIMBC tissues, the miR-155 expression was further measured in 30 matched NIMBC tissues. Cell-free miR-155 levels in urine, as detected by RT-qPCR and RT-qPCR-D, were significantly correlated with their expression in matched tissues (r = 0.763 and 0.728, both at *P* < 0.001; Figure 2B).

### Quantitative analysis of urine cell-free miR-155 using RT-qPCR-D in the validation phase

**Figure 3:**
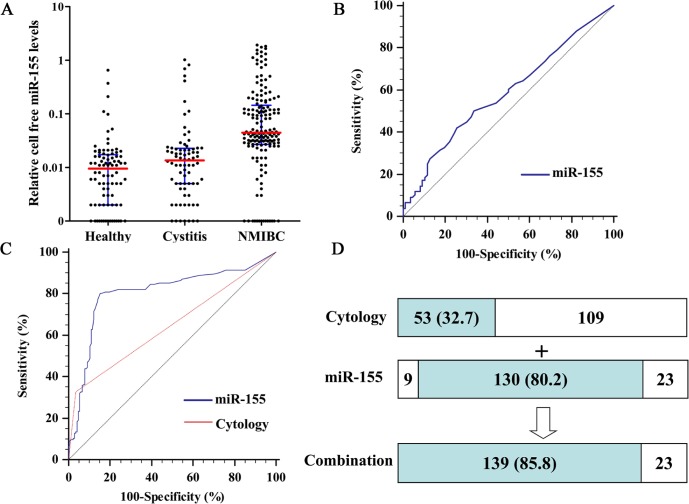
Quantitative analyses of cell-free miR-155 in validation phase and its diagnostic performance **A.** Relative levels of cell-free miR-155 in urine of 162 NIMBC patients, 86 cystitis patients, and 76 healthy donors. **B.** ROC curves for detection of cystitis patients using miR-155. **C.** ROC curves for detection of NMIBC using miR-155 and cytology. **D.** Detection sensitivities for NMIBC of cytology, miR-155 and their combination.

In order to evaluate clinical values of cell-free miR-155 in NIMBC, an established RT-qPCR-D method was used to analyze urine levels of miR-155 in an independent large-scale set of samples. The Kruskal-Wallis test showed a significant difference among 162 NIMBC patients, 86 cystitis patients and 76 healthy donors (*P* < 0.001). As shown in Figure 3A, levels of cell-free miR-155 were significantly elevated in the NIMBC group compared to the cystitis and healthy groups (*P* < 0.001). Moreover, miR-155 levels were significantly higher in the cystitis group than those in healthy group (*P* = 0.038).

### Diagnostic performance of cell-free miR-155 for NIMBC

miR-155 yielded an area under the receiver operating characteristic (ROC) curve (AUC) value of 0.585 (95% CI, 0.505-0.661) in distinguishing cystitis patients from healthy subjects, which showed no significant difference with guessing (*P*=0.060; Figure 3B). When used NIMBC (n = 162) as the end point for detection in comparison to cystitis patients and healthy subjects (n = 162), the AUC for urine miR-155 levels was significantly larger than that for urinary cytology (0.804; 95% CI, 0.756-0.845 versus 0.645; 95%CI, 0.590-0.697, *P* < 0.001; Figure 3C). When the cutoff value was set to the optimal point (0.024, the point on ROC curve corresponding with maximum Youden index (sensitivity+specificity-1) [16]), cell-free miR-155 achieved 80.2% sensitivity and 84.6% specificity. In contrast, urinary cytology had positive detected in 32.7% (53/162) of NIMBC patients within the validation cohort. Combining the urinary cytology with cell-free miR-155 detection increased the number of cases where NIMBC patients were given a positive diagnosis (139/162), and the sensitivity for diagnosing NIMBC increased to 85.8% (Figure 3D).

### Correlation between cell-free miR-155 and clinicopathological characteristics

Associations between cell-free miR-155 and clinicopathological characteristics of NIMBC patients were shown in Table 3. The relative level of cell-free miR-155 was significantly correlated with multifocality (*P* = 0.024), stage (*P* = 0.007) and grade (all at *P* < 0.001). However, no significant associations were found between cell-free miR-155 level and age, gender, tumor size and concomitant carcinoma in situ (CIS) (all at *P* > 0.05). Based on the optimal cut off value (0.024) calculated by ROC analysis, a total of 130 NIMBC patients whose miR-155 levels were above 0.024 were classified as high miR-155 expression group, the remaining 32 cases were classified as low miR-155 expression group. Chi-square tests revealed high cell-free miR-155 expression was only correlated with advanced stage (*P* = 0.013) and high grade (*P* < 0.001).

**Table 3:** Correlations between cell-free miR-155 levels and clinicopathological characteristics

Parameters	cell free miR-155 levels				
	median(interquartile range)	*P*-value^a^	high^c^	low^c^	*P*-value^b^
Age		0.114			0.180
<67 (median)	0.041 (0.025∼0.119)		60	19	
<67	0.046 (0.031∼0.184)		70	13	
Gender		0.215			0.598
Male	0.042 (0.027∼0.136)		100	26	
Female	0.069 (0.033∼0.179)		30	6	
Tumor size		0.343			0.930
<3cm	0.044 (0.026∼0.119)		72	18	
<3cm	0.045 (0.029∼0.251)		58	14	
Multifocality		0.024			0.795
Focal	0.040 (0.028∼0.107)		78	20	
Multifocal	0.095 (0.026∼0.255)		52	12	
Concomitant CIS		0.570			0.050
Yes	0.042 (0.015∼0.343)		24	11	
No	0.045 (0.028∼0.121)		106	21	
Stage		0.007			0.013
Ta	0.032 (0.015∼0.145)		66	24	
T1	0.052 (0.038∼0.151)		64	8	
Grade		<0.001			<0.001
Low	0.029 (0.012∼0.133)		45	23	
High	0.052 (0.036∼0.157)		85	9	

Abbreviations: CIS, Carcinoma in situ.

a *P*-value was estimated by Mann-Whitney U test.

b *P*-value was estimated by Chi-square test.

c Non-muscle invasive bladder cancer patients were classified as high and low cell-free miR-155 groups based on the optimal cut off value (0.024).

### Prognosis value of cell-free miR-155 in NMIBC

**Figure 4:**
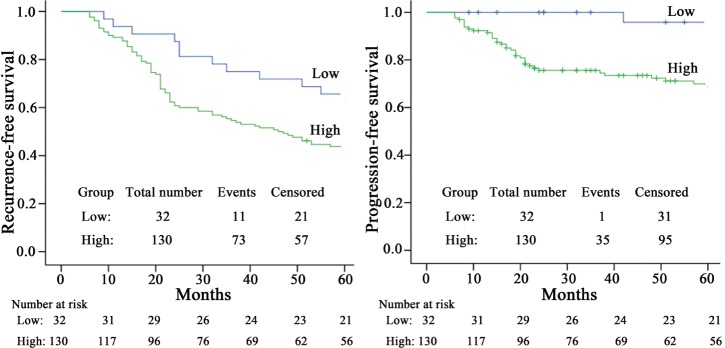
Kaplan-Meier survival curves for NMIBC patients according to miR-155 expression The optimal cutoff value (0.024) of cell free miR-155 was used to categorize the NMIBC patients into high or low level groups.

162 NMIBC patients were followed up with a median duration of 51.5 (range 6-65) months. The 5-year tumor recurrence rate was 51.9% (84/162) and the 5-year progression to muscle invasion rate was 22.2% (36/162). From the Kaplan–Meier curve (Figure 4), patients with high miR-155 levels have dramatically increased tumor recurrence rate and progression to muscle invasion rate than those in the low group (65.6% versus 43.8%, *P* = 0.023 and 95.8% versus 69.9%, *P* = 0.005, respectively). Univariate Cox analysis revealed both recurrence-free survival (RFS) and progression-free survival (PFS) were significantly correlated with concomitant CIS, stage, grade and miR-155 levels (all at *P* < 0.05; Table 4). Significant variables in univariate analyses were evaluated by multivariate analysis to identify the independent factors. CIS, grade and miR-155 maintained their significance as independent prognostic factors for RFS (all at *P* < 0.05; Table 3); whereas concomitant CIS, stage and miR-155 had independent relationships to PFS (all at *P* < 0.05; Table 4).

**Table 4:** Cox proportional hazards regression model analysis of recurrence-free survival and progression-free survival in NMIBC patients

Parameters	Categories	Univariate analysis		Multivariate analysis	
		HR (95% CI)	*P* value	HR (95% CI)	*P* value
**RFS**					
Age	<67 *VS* <67	1.058(0.689∼1.624)	0.797	NA	
Gender	Male *VS* Female	1.465(0.906∼2.368)	0.119	NA	
Tumor size	<3cm *VS* <3cm	1.280(0.834∼1.965)	0.259	NA	
Multifocality	Focal *VS* Multifocal	0.665(0.421∼1.052)	0.081	NA	
Concomitant CIS	Yes *VS* No	5.247(3.321∼8.289)	<0.001	8.445(4.873∼14.636)	<0.001
Stage	Ta *VS* T1	1.937(1.261∼2.977)	0.003	0.861(0.519∼1.428)	0.562
Grade	Low *VS* High	2.892(1.776∼4.712)	<0.001	2.256(1.268∼4.013)	0.006
miR-155	Negative *VS* Positive	2.043(1.083∼3.854)	0.027	3.497(1.722∼7.099)	0.001
**PFS**					
Age	<67 *VS* <67	1.473(0.754∼2.880)	0.257	NA	
Gender	Male *VS* Female	1.462(0.705∼3.034)	0.307	NA	
Tumor size	<3cm *VS* <3cm	1.342(0.698∼2.580)	0.379	NA	
Multifocality	Focal *VS* Multifocal	0.634(0.312∼1.290)	0.209	NA	
Concomitant CIS	Yes *VS* No	4.800(2.423∼9.508)	<0.001	5.420(2.535∼11.586)	<0.001
Stage	Ta *VS* T1	9.927(3.856∼25.554)	<0.001	3.368(1.153∼9.840)	0.026
Grade	Low *VS* High	16.231(3.894∼67.647)	<0.001	4.361(0.858∼22.177)	0.076
miR-155	Negative *VS* Positive	10.146(1.389∼74.091)	0.022	9.466(1.210∼74.066)	0.032

Abbreviations: RFS, Recurrence-free survival; PFS, Progression-free survival; *VS*, versus; HR, Hazard ratio; CI, Confidence interval; CIS, Carcinoma in situ; NA, not considered in the multivariable model.

### Changes of cell-free miR-155 before and after operation in NMIBC patients

**Figure 5:**
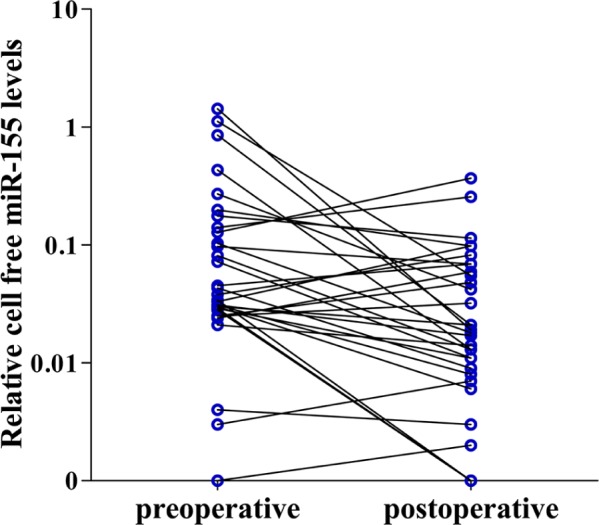
Cell-free miR-155 changes in NMIBC patients before and after operation Cell-free miR-155 was detected in 32 paired preoperative and postoperative (day 14 postoperation) urine samples of NMIBC patients.

As shown in Figure 5, urinary miR-155 levels were reduced in 78.1% (25/32) of NIMBC patients after operation. And, median concentrations of miR-155 in postoperative samples were 0.019(0.008-0.067), which were significantly lower than those in preoperative samples with 0.036(0.028-0.138) (*P*=0.024).

## DISCUSSION

Data from our pilot phase revealed two important observations. First, we established a RT-qPCR-D method to detect cell-free miR-155 in urine supernatant without RNA extraction, which simplified the procedure and reduced the variations. On the clinical level, cell-free miR-155 levels were increased in the urine of NMIBC patients in comparison to healthy controls and a statistically significant correlation was observed between urine and matched tissues, confirming the specificity of miR-155 expression in circulation. Next, in the validation phase, increased miR-155 expression was successfully validated in a large, independent cohort of urine samples. In addition, cell-free miR-155 was significantly decreased after the NMIBC patients underwent transurethral bladder resection. For the first time, we demonstrated that cell-free miR-155 could serve as noninvasive biomarker for NMIBC diagnosis and prognosis prediction.

Previous studies utilizing urine-based miRNAs have mainly focused on urine sediment [8, 9, 17] and the uneven distribution of cellular components might influence reproducibility, limiting the use of miRNA detection as reliable biomarkers. Moreover, miRNAs in urine sediment mainly represented the intracellular expression of renal cells or blood cells while miRNAs in urine supernatant come from the secretion of urinary system tissue cells or circulation via glomerular filtration [18, 19]. Thus, miRNAs in urinary sediment and supernatant might have different expression and different biologic implications [20]. Recently, cell-free miRNAs from serum/plasma have been directly amplified, negating the need for RNA extraction in detecting breast cancer and melanoma, often better reflecting their expression in tissues [21, 22]. More recently, we developed a RT-qPCR based method to detect cell-free mRNAs in colorectal cancer where reverse transcription is performed directly in serum [23]. In the present study, to directly amplify cell-free miRNAs in urine samples, the urine supernatant was first mixed with a preparation buffer containing Tween-20, as the detergent can dissociate the bonds between RNA and protein/lipids/circulating microvesicles meant to decrease degradation [24–26]. Our study demonstrated that the results obtained from RT-qPCR-D were significantly correlated with those detected by RT-qPCR. Moreover, because the direct assay bypassed extraction of circulating RNA from urine, minimizing human and mechanical errors, this method results in decreased inherent variability and has superior reproducibility. Consequently, RT-qPCR-D is a robust and reliable method for the detection of cell-free miRNAs in urine.

Recently, Wang *et al* [20] detected miR-155 level in urine of bladder cancer using traditional RT-qPCR, and found its expression was increased in supernatants while decreased in sediment. The present study used established RT-qPCR-D method and found elevated cell-free miR-155 in urine supernatants of NMIBC patients when compared to cystitis patients and healthy subjects, which was consistent with the previous results that found in urine supernatants [20] and tissues [27, 28] of bladder cancer. Further analyses indicated that advanced grade or stage tumors had higher miR-155 levels. However, the link between miR-155 upregulation and bladder cancer development remains unclear. Recently, Peng *et al* [28] have found that overexpression of miR-155 in bladder cancer cells can promote cell growth, colony formation and cell cycle progression. A variety of studies also provide indirect evidence linking miR-155 expression and bladder cancer, such as, miR-155 overexpression promoting some tumor cell growth via Wnt/β-catenin signaling activation [12, 13] which is also a vital pathway in bladder cancer tumorigenesis [29]. Meanwhile, some tumor suppressor genes have been identified as direct and functional targets of miR-155, such as APC [12], VHL [30], PIK3R1 [31], MLH1 [32], all of where are frequently inactivated in bladder cancer due to promoter hypermethylation [33–36]. Taken together, it appears that miR-155 plays an important role in the initiation and progression of bladder cancer. In our study, urine miR-155 levels were also increased in cystitis patients compared with healthy subjects, consistent with reports that miR-155 is upregulated after T cell activation in response to infectious agents [37, 38]. Tili *et al* [39] suggested that both miR-155 overexpression and inflammatory environments enhance the mutation rate required for tumor development and progression. However, further studies are required to determine if cystitis patients with high miR-155 levels have an increased risk of bladder cancer.

Currently, a variety of commercially available molecular biomarkers are used to diagnose bladder cancer (e.g. ImmunoCyt, NMP22, and BTA stat assays), but none have proven to be ideal or can be recommended without restrictions for NMIBC [40, 41]. These tests are limited by high rates of false-positives and poor sensitivity with low grade tumors [42]. In the present study, ROC analyses demonstrated that the detection performance for miR-155 was more accurate than urine cytology. Detection of urine extracellular miR-155 was robust in distinguishing NMIBC patients from cystitis patients and healthy subjects, with 80.2% sensitivity and 84.6% specificity. Moreover, combining urine cytology and cell-free miR-155 analysis improved the sensitivity from 32.7% to 85.8%. In sum, these results indicate that cell-free miR-155 may be an effective complement to current detection strategies.

Although tumor grade and stage are the most common prognostic factors of bladder cancer, they cannot always predict the biological potential of NMIBC, as this cancer is a heterogeneous spectrum of diseases, and patients at same stage and grade can have completely different clinical outcomes. This study found that cell-free miR-155 expression in urine also served as a prognostic indicator for recurrence and progression to muscle invasion of NMIBC. According to the Kaplan–Meier curve, patients in high urinary miR-155 expression group have dramatically higher tumor recurrence rate and progression to muscle invasion rate than patients in the low group. Cox proportional hazards regressions model confirmed that urinary miR-155 was an independent factor for RFS and PFS-like grade or stage. Wang and Men [27] also reported that elevated miR-155 expression in tissues was associated with poor prognosis of bladder cancer patients. Moreover, urinary miR-155 could be used for disease monitoring since it concentration was significantly decreased after operation. Thus, miR-155 could provide additional prognostic information to inform decisions during disease management.

There are limitations to the methods considered in this study. Currently, the origin and release mechanism of miR-155 present in the urine supernatant is not fully understood, although some investigators suggest that cell-free RNAs are secreted from tissues and as cellular signaling molecules [43, 44]. Based on the matched urine and tissue study, miR-155 expression in urine might reflect, at least partially, its status in NMIBC tissue. In addition, although a combination of urine cytology and cell-free miR-155 analysis increases NMIBC diagnosis sensitivity, further experiments are needed to examine the efficacy of combining miR-155 detection with other common commercially available tests, and how this could be used to reduce reliance on cystoscopic evaluations in follow-up examinations.

In conclusion, this study establishes that the detection of cell-free miR-155 in urine by RT-qPCR-D is a simple and noninvasive method, and could be used for NMIBC diagnosis, as well as predicting recurrence and progression to muscle invasion of NMIBC. Further multi-center studies that include larger cohorts of patients collected from several institutions (including diverse ethnic populations) is required to validate the method's feasibility and clinical utility prior to utilizing the test in routine clinical practice.

## MATERIALS AND METHODS

### Ethics statement

The study was approved by the Ethics Committee of Traffic Hospital of Shandong Province and Qilu Hospital of Shandong University, and written informed consent from all patients was also obtained.

### Study design and patients

NIMBC patients undergoing transurethral bladder resection with confirmed postoperative histopathological analyses were staged, and graded according to 2010 AJCC/TNM classification and 2004 WHO/ISUP criteria, respectively. CIS were examined by random biopsies and then confirmed by pathologists. And, the NMIBC patients with controversial diagnosis were not included in this study. Healthy controls were collected from subjects who had normal laboratory results from routine checkups. Subjects with histories of cancer were excluded from this study.

This study was designed as both a pilot phase and a subsequent validation phase. In the pilot phase, urine samples were collected from 30 NIMBC patients and 30 sex/age-matched healthy subjects that were recruited from the Shandong Traffic Hospital to determine the feasibility of the RT-qPCR-D method. Additionally, tumor tissues from the above NIMBC patients were available to examine a correlation between miR-155 levels in urine and tissue samples. In the validation phase, changes of cell-free miR-155 levels in urine supernatants were validated in a large, independent cohort of 162 NIMBC patients, 76 cystitis patients, and 86 healthy donors enrolled from Qilu Hospital, Shandong University. Among them, NIMBC patients were followed up with cystoscopy quarterly for the first 2 years, then semiannually for 5 years, and annually thereafter until January 2014. We defined recurrence and progression to muscle invasion as examination by histologically confirmed tumors and tumors staged T2 or higher in the bladder cancer after treatment during follow-up. In addition, paired preoperative and postoperative (day 14 postoperation) urine samples were collected from another independent cohort of 32 NIMBC patients undergoing transurethral bladder resection in Qilu Hospital, Shandong University for disease monitoring.

### Sample processing

Freshly-voided urine samples were collected in the morning before cystoscopy examination and any treatments, and the samples were centrifuged at 3000 xg for 10 min. The cellular pellet was used for cytology analysis and supernatant was further centrifuged at 16,000 xg for 10 min at 4°C and then stored at −80°C until use. Tumor tissues selected for RNA extraction were immediately stored in liquid nitrogen and the remaining tissues were fixed in formaldehyde for pathological analysis. The postoperative histopathological analyses and urine cytology tests were performed with hematoxylin and eosin staining and reviewed to confirm the diagnosis by 2 experienced pathologists.

### RNA extraction

RNA was extracted from tissues using the standard TRIzol method (Invitrogen, Carlsbad, CA) following the manufacturer's protocol. For urine samples, RNA was isolated with miRNeasy Serum/Plasma Kit (Qiagen, Valencia, CA). In brief, 200 μl of urine supernatant was lysed in 5 volumes of Qiazol solution, then the mixture was processed according to the manufacturer's protocol, and RNA was eluted using 14 μl of RNase-free water. The concentration of RNA was measured using a NanoDrop spectrophotometer (Thermo Fisher Scientific, Waltham, MA).

### RT-qPCR with extracted RNA

RT-qPCR was performed in an ABI Prism 7500 System (Applied Biosystems) using a SYBR PrimeScript miRNA RT-qPCR Kit (Takara, Dalian, China). The miRNA-specific forward primers this study used were the forward qPCR primers of Bulge-loop™ miRNA qRT-PCR Primer Sets (containing one RT primer, a pair of qPCR primers for each set) specific for miR-155, U6 and RNU48, which were designed by RiboBio (Guangzhou, China). And Uni-miR qPCR Primer (one reagent in SYBR PrimeScript miRNA RT-qPCR Kit) was used as reverse primer according to manufacturer's instructions. In brief, 50 ng of RNA (including miRNAs) was first polyadenylated and reverse-transcribed with a poly(T) adapter into cDNAs in triplicates in a final volume of 20 μl. The conditions for reverse transcription were 37°C for 60 min and 85°C for 5 s. Then, 5 μl of 10-fold diluted cDNA were added to a qPCR reaction containing 12.5 μl of SYBR Premix Ex Taq II, 0.5 μl of Dye II, 1 μl of Uni-miR qPCR Primer, and 1 μl of forward primer for U6, RNU48 or miR-155and 5 μl of ddH_2_O. The amplification was performed in duplicate as follows: 95°C for 10 min, 40 cycles of 95°C for 15 s and 60°C for 1 min, and melting curve analysis.

### RT-qPCR directly applied in urine (RT-qPCR-D)

The 2X Preparation Buffer contained 1.5% Tween 20 (EMD Chemicals, Gibbstown, NJ), 50 mM Tris (Sigma-Aldrich, St. Louis, MO) and 1 mM EDTA (Sigma-Aldrich, St. Louis, MO). First, 3 μl of urine supernatants were mixed with an equal volume of 2X Preparation Buffer and were then reverse transcribed in triplicate in a final reaction volume of 20 μl. A 1:10 dilution of the reverse transcribed product was then centrifuged at 16,000 xg for 5 min and 5 μl supernatant solution was used as a cDNA template for qPCR. The reagents and reaction conditions were the same as RT-qPCR.

### Primary data collection and data normalization

The formula (PAE+1)^−ΔCq^ (ΔQuantification cycle (Cq)=[Cq(test sample)-Cq(calibrator sample)] was used for target gene calculation. T24 bladder cancer cell was used as calibrator sample in each run for eliminating the inter-assay variation. The levels of tissue miR-155 were normalized using RNU6B, as recommended in a variety of other studies. The relative gene expression level in each urine sample was recorded as the ratio of miR-155 expression to the geometric mean of U6 and RNU48, as previously described [45].

### Statistical analysis

Difference between miR-155 levels among multiple groups was determined by the Kruskal-Wallis test and further comparisons between the two groups were evaluated using the Mann–Whitney U test. The Spearman correlation coefficient examined associations between RT-qPCR-D and RT-qPCR methods, as well as between urine and tissue samples. Survival curves were constructed by the Kaplan-Meier method and a log-rank test was used to compare the distributions of survival times. The Cox model was employed to evaluate the independent factors of RFS and PFS. Changes of cell-free miR-155 before and after operation were compared by Wilcoxon matched-pairs signed-ranks test. These statistical analyses were performed using SPSS Statistics 17.0 for Windows (IBM Corporation, Armonk, NY). To illustrate the diagnostic power of the tests, ROC curves were established and the AUCs with 95% CI were compared by the method of Delong *et al* [46] using MedCalc 9.3.9.0 (MedCalc, Mariakerke, Belgium). Statistical significance was defined as a two-sided *P* value of less than 0.05.
